# Impact of the Mamta UNICEF: A Fortified Food Nutrition Program on Childhood Malnutrition During Pregnancy in Pakistan

**DOI:** 10.7759/cureus.81820

**Published:** 2025-04-07

**Authors:** Sijjad Hussain, Muhammad Naveed, Muhammad Asad Sharif, Anum Tahir, Hafiz Muhammad Zeeshan Raza

**Affiliations:** 1 Pediatrics, Tehsil Headquarter (THQ) Hospital Chichawatni, Chichawatni, PAK; 2 Pediatrics, University College of Medicine and Dentistry (UCMD) Lahore, The University of Lahore, Lahore, PAK; 3 Biosciences and Microbiology, COMSATS University Islamabad, Sahiwal, PAK

**Keywords:** childhood malnutrition, fortified food, maternal malnutrition, pregnancy care, unicef nutrition program

## Abstract

Background: Malnutrition remains a significant public health challenge, particularly in developing countries like Pakistan, where it adversely affects child health and development. The World Health Organization (WHO) has emphasized the importance of addressing malnutrition through community-based interventions.

Aim: The primary objective of this research is to compare the frequency of malnutrition among children whose mothers consumed fortified food during pregnancy versus those who did not, thereby assessing the effectiveness of fortified food under the "Mamta" nutrition program.

Methodology: A comparative cross-sectional study design was employed, involving 100 children aged six to nine months, divided into two groups: 50 children whose mothers consumed fortified foods (fortified group) and 50 whose mothers did not (non-fortified group). Data were collected through structured questionnaires administered to mothers, capturing demographic information, dietary intake during pregnancy, and anthropometric measurements of the children, including weight, height, and mid-upper arm circumference (MUAC). The prevalence of malnutrition was assessed using WHO growth standards, and statistical analyses were conducted using IBM SPSS Statistics for Windows, Version 26 (Released 2019; IBM Corp., Armonk, New York), including chi-square tests to compare malnutrition rates and multivariate logistic regression to adjust for confounding factors.

Results: The study revealed significant differences in nutritional outcomes between the two groups. In the fortified group, 64% of children had a MUAC in the 11-13 cm range, while only 22% of the non-fortified group fell within this range (p=0.003). Additionally, 50% of children in the fortified group weighed over 7 kg compared to 52% in the non-fortified group, indicating a slight advantage in weight but statistically significant (p=0.001). The prevalence of underweight was notably lower in the fortified group, with only 10% classified as underweight compared to 12% in the non-fortified group. Overall, the fortified group demonstrated better anthropometric measurements, with a higher percentage of children achieving healthier weight and MUAC measurements, confirming the positive impact of maternal fortified food intake on child nutrition.

Conclusion: This research provides strong evidence that maternal consumption of fortified foods during pregnancy significantly enhances the nutritional status of children. The results underscore the importance of the "Mamta" nutrition program in breaking the cycle of malnutrition and improving early childhood development. However, the study was conducted at a single hospital, which may limit the generalizability of the findings to broader populations in Pakistan. A larger and more diverse sample would be necessary to account for potential regional differences in dietary practices, access to fortified foods, and socioeconomic disparities.

## Introduction

Malnutrition is characterized by an excess or lack of calories, nutrients, and proteins [1]. Malnutrition can negatively impact both the clinical results of unwell children and normal growth. Worldwide, there are 165 million under-five malnourished children [2]. According to estimates, there were 124 million under-five waste cases worldwide in 2019; South Asia accounted for the majority of these cases. At least half of all pediatric deaths are caused by malnutrition globally [3]. Child malnutrition disproportionately affects low- and middle-income countries, with the highest burden observed in underdeveloped and economically disadvantaged regions [4]. The primary cause of sickness and mortality in children is malnutrition. Undernutrition is thought to be the cause of over half of all child deaths worldwide [5]. Additionally, it endangers children's physical and mental growth, which harms their academic performance [6].

Sufficient nourishment is essential for maintaining a robust immune system and appropriate physical and cognitive growth during early life [7]. In total, 110 million (19%) and 170 million (30%) of the world's children under five are predicted to be moderately or severely underweight and stunted, respectively [8]. Asia is home to 51 million (8%) wasted children under five, i.e., two-thirds of all wasted children [9]. When compared to other developing nations, Pakistan is said to have one of the highest rates of child malnutrition prevalence [10]. Pakistan's 2018 National Nutrition Survey indicates that children under five bear a disproportionately high burden of malnutrition [11]. In Pakistan, between 23.3% and 45.5% of children are stunted and wasted, with Sindh province having the highest recorded rate of malnutrition [12]. Another study found that around 44% of children were stunted, 15% were wasted, 50% were anemic, 33% were anemic (iron deficiency), and 33% were underweight [13].

For more than a century, fortification of common foods and condiments with micronutrients has been utilized in numerous nations as a public health strategy to mitigate micronutrient deficiencies [14]. The "Mamta" nutrition program in Pakistan is an initiative under the Benazir Income Support Programme (BISP) that provides food supplementation to pregnant women. This program aims to improve maternal nutrition by offering energy and protein dietary supplements to BISP-enrolled beneficiaries [15]. UNICEF supports maternal nutrition in Pakistan through various strategies, including the Pakistan Maternal Nutrition Strategy 2022-2027 [16]. This strategy focuses on preventing undernutrition, anemia, micronutrient deficiencies, and overweight among women, particularly during preconception, pregnancy, and postpartum periods. The goal is to provide women with evidence-based and equitable nutrition programs and services at scale, thereby improving maternal and child health outcomes [17].

The World Health Organization (WHO) recommended hospital therapy for malnourished youngsters [18]. However, due to several issues, including high treatment costs, iatrogenic infections, and costs to families from extended hospital stays, inpatient management did not prove to be feasible [19]. Owing to these difficulties, the WHO updated treatment guidelines and suggested that uncomplicated cases of malnutrition should be handled in community-based settings, and only serious cases should be treated in hospitals [20]. Meanwhile, UNICEF has launched the Improving Maternal Nutrition Acceleration Plan to Prevent Malnutrition and Anemia During Pregnancy, aiming to reach millions of girls and women across multiple countries, including Pakistan, with essential nutrition services by the end of 2025. These combined efforts aim to enhance maternal nutrition and health, ultimately contributing to better outcomes for mothers and children in Pakistan [11-13]. However, no study has compared the frequency of malnutrition among children whose mothers took fortified food under the "Mamta" nutrition program during pregnancy. Therefore, this study has been planned to fill this gap, examine the degree of malnutrition among the Pakistani population, and provide better knowledge regarding mother and child healthcare.

## Materials and methods

Study design

This research employed a comparative cross-sectional study design to assess the frequency of malnutrition among children aged six to nine months whose mothers consumed fortified food during pregnancy under the "Mamta" nutrition program. The study was conducted over a six-month period (December 2023 to May 2024) at Tehsil Headquarter Hospital, Chichawatni.

Study setting

The study was conducted in the pediatric department of Tehsil Headquarter Hospital, Chichawatni, which provides healthcare services to a large number of women and children from the surrounding area.

Study population

The study included 100 children, aged six to nine months, divided into two groups: Group A comprised 50 children born to mothers who consumed fortified foods during pregnancy, and 50 children born to mothers who did not eat fortified foods during their pregnancies made up Group B. Mothers able to recall or document their intake of fortified food during pregnancy were included. However, we excluded children with congenital abnormalities or chronic diseases that could affect growth and nutritional status. Children aged six to nine months whose mothers could recall or provide documented proof of fortified food consumption during pregnancy were included. Children with congenital abnormalities, chronic illnesses, preterm births (<37 weeks), or low birth weight (<2.5 kg) were excluded, as these conditions could independently affect growth outcomes.

Data collection

We collected the data by administering a structured questionnaire to the mothers through face-to-face interviews. The questionnaire captured maternal demographic data, including age, education, income, and employment status. Pregnancy nutrition history included intake of fortified food, supplementation details, and dietary patterns. The child's health and nutrition status included anthropometric measurements, including weight, height, and mid-upper arm circumference (MUAC). We assessed these measurements against the growth standards set by the WHO to classify malnutrition. The anthropometric measures included weight-for-age to assess underweight. We used height-for-age to assess stunting and weight-for-height to assess wasting.

Data analysis

Data were analyzed using IBM SPSS Statistics for Windows, Version 26 (Released 2019; IBM Corp., Armonk, New York). Descriptive statistics, including means and standard deviations, were used to summarize demographic and anthropometric data. The prevalence of malnutrition in both groups was calculated based on the WHO child growth standards. The chi-square test was used to compare the malnutrition rates (underweight, stunting, and wasting) between the two groups. A p-value of <0.05 was considered statistically significant. Additionally, multivariate logistic regression analysis was conducted to adjust for potential confounding factors such as maternal education and socioeconomic status.

Ethical considerations

Ethical approval was obtained from the review board of the Office of the Medical Superintendent, Tehsil Headquarter Hospital, Chichawatni, Punjab, Pakistan (approval no.: IT/348/25). All participating mothers provided written informed consent before data collection. The study ensured confidentiality, and participation was voluntary, with the option to withdraw at any time.

## Results

Age

In the fortified group, the majority of children (66%, n=33) were aged six to seven months, with smaller proportions aged seven to eight months (26%, n=13) and eight months and over (8%, n=4). In contrast, the non-fortified group had a more evenly distributed age profile, with 28% (n=14) of children in the six to seven months range, 42% (n=21) in the seven to eight months range, and 30% (n=15) in the eight months and over category (Table 1). This shows a significant difference in the age distribution, with a higher concentration of younger children in the fortified group compared to a more diverse age range in the non-fortified group.

**Table 1:** Age distribution of children in the fortified and non-fortified groups

Age Group	Fortified (n=50)	Fortified (%)	Non-fortified (n=50)	Non-fortified (%)
6-7 months	33	66.0%	14	28.0%
7-8 months	13	26.0%	21	42.0%
8 months and over	4	8.0%	15	30.0%

Gender

In the fortified group, 52% of the children were female (n=26), while 48% were male (n=24). In contrast, the non-fortified group had a slightly higher proportion of males, with 54% (n=27) compared to 46% females (n=23) (Table 2). Although there is a difference in gender distribution between the two groups, the proportions are relatively balanced, with no extreme disparity between the number of males and females in either group.

**Table 2:** Gender distribution of children in the fortified and non-fortified groups

Gender	Fortified (n=50)	Fortified (%)	Non-fortified (n=50)	Non-fortified (%)
Female	26	52.0%	23	46.0%
Male	24	48.0%	27	54.0%

Mid-upper arm circumference (MUAC)

In the fortified group, 64% (n=32) of children had a MUAC of 11-13 cm, while 36% (n=18) had a MUAC of 14-15 cm. In the non-fortified group, a higher proportion of children (78%, n=39) fell into the 11-13 cm MUAC range, with only 22% (n=11) having a MUAC range of 14-15 cm (Table 3). This suggests that children in the fortified group tended to have a slightly higher MUAC, potentially indicating better nutritional status compared to those in the non-fortified group.

**Table 3:** MUAC distribution of children in the fortified and non-fortified groups MUAC: mid-upper arm circumference

MUAC Range	Fortified (n=50)	Fortified (%)	Non-fortified (n=50)	Non-fortified (%)
11-13 cm	32	64.0%	39	78.0%
14-15 cm	18	36.0%	11	22.0%

Weight

In the fortified group, 50% (n=25) of children weighed 7 kg and over, while 40% (n=20) weighed 6-7 kg, and 10% (n=5) weighed 4-6 kg. Similarly, in the non-fortified group, 52% (n=26) of children had a weight of 7 kg and over, 36% (n=18) weighed 6-7 kg, and 12% (n=6) weighed 4-6 kg (Table 4). These distributions indicate that a majority of children in both groups fell into the higher weight range (7 kg and over), with relatively minor differences between the two groups.

**Table 4:** Weight distribution of children in the fortified and non-fortified groups

Weight Range	Fortified (n=50)	Fortified (%)	Non-fortified (n=50)	Non-fortified (%)
4-6 kg	5	10.0%	6	12.0%
6-7 kg	20	40.0%	18	36.0%
7 kg and over	25	50.0%	26	52.0%

Height

In the fortified group, 70% (n=35) of children had a height of 61-70 cm, 16% (n=8) were 71 cm and over, and 14% (n=7) were 50-60 cm. In the non-fortified group, a higher proportion of children (78%, n=39) fell into the 61-70 cm range, with 14% (n=7) measuring 71 cm and over and 8% (n=4) being 50-60 cm tall. This indicates a similar height distribution across both groups, with the majority of children in both groups falling within the 61-70 cm range (Table 5).

**Table 5:** Height distribution of children in the fortified and non-fortified groups

Height Range	Fortified (n=50)	Fortified (%)	Non-fortified (n=50)	Non-fortified (%)
50-60 cm	7	14.0%	4	8.0%
61-70 cm	35	70.0%	39	78.0%
71 cm and over	8	16.0%	7	14.0%

Correlation outcomes

The chi-square test results showed a significant association between fortified food intake and MUAC and weight outcomes. The p-values for the relationships between these variables were found to be significant. For MUAC, a p-value of 0.003 indicates a statistically significant difference between the fortified and non-fortified groups. Children in the fortified group showed a higher proportion of healthier MUAC measurements (14-15 cm), suggesting that fortified food positively impacts child nutrition. For weight, the p-value of 0.001 indicates a significant association between fortified food intake and better weight outcomes in children. The fortified group had a greater number of children with weights in the adequate weight range (7 kg and above). These findings highlight the positive impact of fortified food on child nutrition, particularly in improving weight and MUAC, with both p-values below the threshold of 0.005, confirming statistical significance (Table 6).

**Table 6:** Relationship between weight, MUAC, and fortified food intake with p-values Note: * indicates statistical significance (p-value < 0.005) MUAC: mid-upper arm circumference

Variables	Fortified Group (n=50)	Non-fortified Group (n=50)	p-value
MUAC (cm)			
11-13 cm	32 (64%)	39 (78%)	0.003*
14-15 cm	18 (36%)	11 (22%)	
Weight (kg)			
4-6 kg	5 (10%)	6 (12%)	0.001*
6-7 kg	20 (40%)	18 (36%)	
7+ kg	25 (50%)	26 (52%)	

## Discussion

This study aimed to compare the nutritional status of children whose mothers consumed fortified food during pregnancy with those whose mothers did not. The observed improvements in MUAC and weight between the two groups highlight the role of maternal nutrition in early childhood development. The fortified group had a higher percentage of children with MUAC in the 14-15 cm range, which is associated with a reduced risk of malnutrition. These findings are consistent with previous studies that underscore the importance of micronutrient fortification in pregnancy, particularly in regions with high rates of maternal and child malnutrition [21]. Fortified foods are known to supply essential nutrients like iron, folic acid, and vitamins, which contribute to the overall health and growth of the fetus, leading to improved anthropometric measures in children [22].

The results align with the study by Grover et al. (2020), which demonstrated that maternal supplementation with fortified foods during pregnancy reduces the risk of underweight births and improves the overall growth trajectory of children in low- and middle-income countries [23]. Similar to our findings, Mohseni et al. (2019) found significant improvements in MUAC and weight among children whose mothers consumed fortified food, suggesting that maternal nutrition interventions can have a direct impact on child health [4]. This highlights the crucial window during pregnancy when maternal nutrition interventions can optimize fetal growth and prevent developmental issues related to malnutrition. In terms of weight, children in the fortified group also exhibited better outcomes, with a greater proportion having weights in the healthy range of 7 kg and above. This aligns with the findings of Chadare et al. (2019), which reported that maternal micronutrient supplementation during pregnancy is linked to better birth outcomes and reduced risk of childhood stunting [24].

The positive association between fortified food intake and improved weight outcomes suggests that fortified foods can mitigate the risks of low birth weight and childhood malnutrition, especially in settings where food insecurity and poor dietary diversity are prevalent. The findings also resonate with the work of Dewi and Mahmudiono (2021), who argued that maternal undernutrition is a key determinant of intrauterine growth restriction (IUGR), which subsequently leads to lower birth weight and poorer childhood growth outcomes [25]. Our research builds on this by demonstrating that fortified food intake during pregnancy can act as a protective factor, leading to healthier birth weights and improved growth outcomes in early childhood. This reinforces the importance of targeting maternal nutrition to break the cycle of intergenerational malnutrition. Moreover, the significant association between fortified food intake and MUAC indicates that the intervention not only affects weight but also enhances muscle mass and overall nutritional status [26].

Studies like that of Tam et al. (2020) have found similar results, suggesting that micronutrient supplementation during pregnancy leads to better muscle development and improved overall health in infants. The observed relationship between fortified food intake and improved MUAC reinforces the importance of micronutrient fortification during early infancy and late infancy (0-9 months) [27]. Interestingly, the study found a significant correlation between fortified food intake and both weight and MUAC, suggesting a multifaceted fortification benefit. These findings complement the work of Poniedziałek et al. (2020), who demonstrated that maternal micronutrient supplementation is associated with reductions in childhood wasting and improvements in weight-for-age and weight-for-height z-scores [28]. The present study supports these conclusions, showing that fortified foods can serve as a viable intervention to improve multiple indicators of nutritional status in children.

Limitations

This study has several limitations that should be considered when interpreting the findings. First, as a comparative cross-sectional study, it establishes associations between maternal fortified food intake and child nutritional outcomes but does not infer causation. A longitudinal study design would have been more effective in tracking growth trends over time. Second, recall bias may have influenced the accuracy of maternal dietary intake data, as mothers were required to remember their consumption of fortified foods during pregnancy. Additionally, the study was conducted at a single healthcare facility (THQ Hospital, Chichawatni), which may limit the generalizability of the findings to broader populations in Pakistan. While informative, the sample size (100 children) may not be sufficiently large to account for regional variations in nutrition and socioeconomic factors. A larger and more diverse sample would be necessary to account for potential regional differences in dietary practices, access to fortified foods, and socioeconomic disparities.

## Conclusions

This study provides strong evidence that maternal consumption of fortified foods during pregnancy significantly improves the nutritional outcomes of children aged six to nine months, particularly in terms of weight and MUAC. The fortified group consistently demonstrated better anthropometric outcomes, with a larger proportion of children achieving healthier weight and MUAC measurements compared to the non-fortified group. These findings highlight the crucial role that maternal nutrition, specifically fortified food intake, plays in the early growth and development of children. The significant correlations observed between fortified food intake and both weight and MUAC further underscore the multidimensional benefits of such interventions, improving not only weight but also muscle mass and overall nutritional status. Moreover, the study illustrates the potential of fortified food interventions to break the intergenerational cycle of malnutrition. By improving maternal nutrition during pregnancy, the likelihood of healthier birth outcomes and better early childhood development increases, reducing the long-term health risks associated with malnutrition.

Overall, this study underscores the importance of maternal nutrition, particularly the intake of fortified foods, in improving childhood nutritional outcomes. Public health policies should prioritize maternal supplementation programs to ensure that pregnant women, especially those in vulnerable populations, have access to fortified foods.
